# Three-Dimensional Printing of Triboelectric Nanogenerators by Digital Light Processing Technique for Mechanical Energy Harvesting

**DOI:** 10.1021/acsami.3c13323

**Published:** 2023-11-09

**Authors:** Annalisa Chiappone, Ignazio Roppolo, Edoardo Scavino, Giorgio Mogli, Candido Fabrizio Pirri, Stefano Stassi

**Affiliations:** † Department of Chemical and Geological Sciences, Università degli studi di Cagliari, Cittadella Universitaria Blocco D, S.S. 554 bivio per Sestu, Monserrato, CA 09042, Italy; ‡ Department of Applied Science and Technology, Politecnico di Torino, C.so Duca degli Abruzzi 24, Turin 10129, Italy; § Center for Sustainable Future Technologies @Polito, Istituto Italiano di Tecnologia, Via Livorno, 60, Turin 10144, Italy

**Keywords:** 3D printing, DLP, triboelectric nanogenerator, triboelectric series, energy harvester

## Abstract

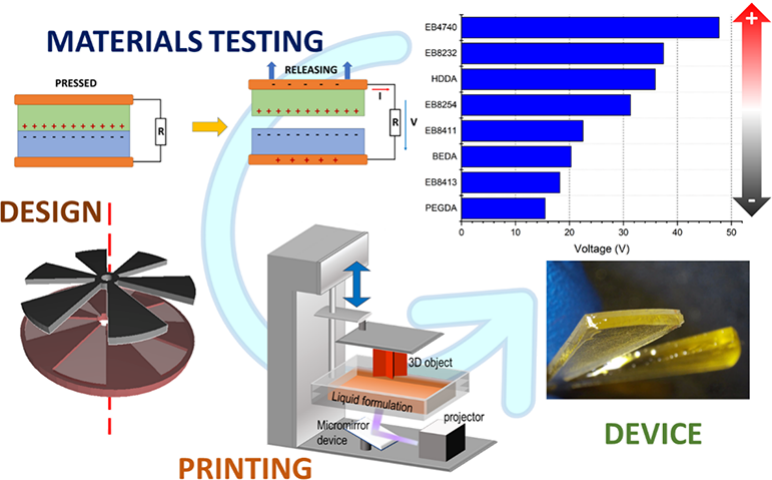

Triboelectric nanogenerators (TENGs) represent intriguing technology to harvest human mechanical movements for powering wearable and portable electronics. Differently, compared to conventional fabrication approaches, additive manufacturing can allow the fabrication of TENGs with good dimensional resolution, high reproducibility, and quick production processes and, in particular, the obtainment of complex and customized structures. Among 3D printing technologies, digital light processing (DLP) is well-known for being the most flexible to produce functional devices by controlling both the geometry and the different ingredients of printable resins. On the other hand, DLP was not exploited for TENG fabrication, and consequently, the knowledge of the performance of 3D printable materials as charge accumulators upon friction is limited. Here, the application of the DLP technique to the 3D printing of triboelectric nanogenerators is studied. First, several printable materials have been tested as triboelectric layers to define a triboelectric series of DLP 3D printable materials. Then, TENG devices with increased geometrical complexity were printed, showcasing the ability to harvest energy from human movement. The method presented in this work illustrates how the DLP may represent a valuable and flexible solution to fabricate triboelectric nanogenerators, also providing a triboelectric classification of the most common photocurable resins.

## 1 Introduction

Modern technological developments require capillary diffusion of electrical devices in daily life, which, in turn, need continuous power supply, provided by an electric grid or by storage systems, such as batteries or supercapacitors.^1^ Consequently, together with the diffusion of technology, the energy demand becomes more and more pivotal. This fast-growing request is nowadays covered by a massive use of fossil fuels, leading to impactful consequences on the environment and lives, as witnessed by pollution measurements and climate changes.

It is thus necessary to focus research efforts on new and sustainable sources in order to achieve environmental objectives and to mitigate the effects of past actions. The exclusive use of energy from renewable sources, such as sunlight, wind, and waves, has been proven not to be suitable in a short amount of time due to infrastructural issues and difficulties related to their discontinuous nature. Obtaining a fully renewable energy system capable of handling a continuous demand will be necessary to install many other infrastructures for production and also for distribution and temporary storage.^2^ Another main concern is the dependency from the storage systems: with the constant improvement of the skills and the computing power of portable devices, it implies an enormous request for batteries or supercapacitors, which in turn leads to other problems, such as materials supply. Additionally, the recent development of wearable flexible electronics is strongly hindered by the necessity to use rigid batteries or to design a wired supply, which affects comfort and performances.^3,4^

An interesting alternative to power portable devices consists of harvesting mechanical energy from body movements, a green alternative that allows the users to be far from the power grid for a potentially unlimited period, free to use everywhere personal devices that normally can work for just some hours.^5^ Ideal harvesters for portable devices should be lightweight, flexible, durable, and dimensionally scalable. It is also important that their performance remains stable with discontinuous energy input.^6^

In the last 20 years, nanogenerators (NG) emerged as an intriguing solution that can match all these requirements. The NGs can convert low intensity discontinuous mechanical stimuli, such as movements in the human body, into electrical power. Their implementation can effectively harvest the kinetic and mechanical energy that is normally wasted in daily activities, from walking to simply typing on a keyboard.^7,8^ The integration of NGs into our personal devices could ideally obtain self-powered systems capable of working with a clean energy source. In this frame, since their first introduction in 2006 by Wang and Song,^9^ NGs have been studied, exploiting first the piezoelectric effect (PENGs)^10−12^ and then the triboelectric effect (TENGs).^13,14^

The latter are now gaining popularity, since those are more convenient compared to piezoelectric materials in terms of cost of production, efficiency, and environmental sustainability.^15,16^ Furthermore, the most important strength of the TENGs over the other NGs is that they are based on the triboelectric effect, consisting of transfer charges when rubbing two different materials, thus occurring theoretically between every couple of materials that come in contact.^17^ However, the amount of electrical power generated depends on the materials characteristics, principally, the capacity of generating charges on the contact surface upon friction as well as the type of charge accumulated. Those properties are well studied in the literature and summarized in a list known as triboelectric series, where the position of each material determines its efficiency as TENG.^18^ Triboelectric series are crucial to make a correct materials’ design to compose a TENG, fulfilling both mechanical and functional properties required, e.g., to obtain lightweight, flexible, stretchable, washable, and even biointegrable TENGs.^19^

As mentioned, the range of materials that can be used for TENGs is virtually unlimited, and consequently, many different fabrication techniques have been exploited. In this context, a key factor in the development of TENGs is the possibility of fabricating devices with good dimensional resolution, high reproducibility, and quick production processes. All these characteristics well match with addictive manufacturing technologies, which allowed the production of different TENG structures with geometrical complexity, difficult to obtain with conventional fabrication approaches.^20,21^ Many different 3D printing technologies, such as fused filament fabrication (FFF, also known as fused deposition modeling, FDM),^22,23^ direct ink writing (DIW),^24,25^ selective laser sintering (SLS),^26^ and stereolithography (SLA),^27^ have been already implemented for the fabrication of triboelectric nanogenerators.

Among all these technologies, vat-photopolymerization (VP) 3D printing, which encompasses SLA and also digital light processing (DLP), are particularly interesting, since those are well-known for being the most flexible techniques to produce functional devices. In fact, in VP is possible to control the functionality both working on the design and on the composition of the printable formulations.^28,29^ Furthermore, these techniques are characterized by very high resolution^30^ as well as the possibility to print multimaterial structures.^31,32^ Among the vat-polymerization 3D printing technology, DLP offers the further advantage of higher printing velocity compared to SLA, since a complete layer of material is cured at the same time.^33^

To the best of our knowledge, only Yoon et al.^34^ reported of a device partially fabricated with DLP able to harvest mechanical energy, employing photocurable resins mixed with PTFE powders. In this work, a complex structure was produced to obtain an air filter which also possesses triboelectric properties. In particular, the proposed TENG generates energy thanks to the flow of air through meshes inserted in the 3D printed structure. Although this device has 2-fold functionality, as air-filter and TENG, the obtained energy conversion performances were not pivotal in the research. Moreover, only a part of the device was fabricated with a 3D printed DLP approach, while the whole TENG fabrication needed further steps, not employing 3D printing technology. With this field being almost unexplored, the main limitation in the use of DLP for TENG fabrication appears related to the limited knowledge of the triboelectric performance of 3D printable materials as charge accumulators upon friction since those are not present in triboelectric series.

To fulfill this lack, in this work, the application of digital light processing techniques for the 3D printing of triboelectric nanogenerators is studied. First, several of the most common printable materials have been characterized to evaluate their performances as triboelectric layers and to define a triboelectric series of DLP 3D printing exploitable materials. Then, TENGs devices with an increase in geometrical complexity have been printed using materials on the opposite side of this triboelectric series. These TENGs represent the first examples of mechanical energy harvesting completely fabricated with photocurable resins with the DLP approach. Mechanical energy from biometric movement was harvested from 3D printed TENGs, which were able to generate electrical power in line with TENGs fabricated with other 3D printing techniques. The comprehensive approach presented in this work demonstrates the capability of the DLP approach to be implemented for triboelectric nanogenerator fabrication and provides a triboelectric classification of the most common photocurable resins, a fundamental starting point to develop future research works in the field.

## 2 Materials and Methods

### 2.1 Materials

The acrylate polydimethylsiloxane TegoRAD 2800 (TEGORAD) was kindly provided by Evonik industries AG (Essen, Germany). Acrylate resins among which bisphenol A ethoxylate diacrylate (BEDA), 1,6-hexanediol diacrylate (HDDA), and poly(ethylene glycol) diacrylate with an *M*_n_ of 700 (PEGDA) were from Sigma-Aldrich. Acrylate urethanes EBECRYL 4740 (EB4740), EBECRYL 8232 (EB8232), EBECRYL 8254 (EB8254), EBECRYL 8411 (EB8411), and EBECRYL 8413 (EB8413) were kindly provided by Allnex GmbH (Germany). Depiction of the chemical formula (where available) and more information are reported in Table S1 in the Supporting Information. Phenyl bis(2,4,6-trimethylbenzoyl)-phosphine oxide (BAPO) and 2-hydroxy-2 methylpropiophenone (HMP), used as photoinitiators, were purchased from Sigma-Aldrich. Propylene carbonate (Sigma-Aldrich) was employed as solvent to solubilize photoinitiator in EBECRYL 8413. Ethanol (99.8% pure) used for 3D printed structures cleaning and chloroform (99% pure), employed to remove soluble fraction of samples, were supplied by Sigma-Aldrich too.

### 2.2 3D Printable Ink Preparation

3D printable inks were prepared solubilizing into acrylate resins (TEGORAD, BEDA, HDDA, PEGDA, EB4740, EB8232, EB8254, EB8411, and EB8413) with different types of photoinitiators. BAPO was employed as photoinitiator for BEDA, HDDA, PEGDA, EB4740, EB8232, and EB8254 inks. HMP was used as the photoinitiator for both EB8411 and EB8413. In the latter, the presence of a solvent was necessary to reduce the viscosity of the urethane resin, enabling the HMP solubilization. Therefore, propylene carbonate was added in a weight ratio of 1:1 with EB8413. In this way, a homogeneous solution was achieved. Regarding TEGORAD, BAPO was first solubilized in HMP with a weight ratio of 1:4 and then the blend was added in the monomer.

All formulations were prepared by mixing 2 wt % of photoinitiator (or photoinitiators blend) through a centrifugal mixer (THINKY Mixer ARE-250) at 1200 rpm for 8 min. Then, a defoaming process (400 rpm for 8 min) was applied to remove air bubbles. Formulations containing EBECRYL were preheated at 65 °C using a laboratory oven (MEMMERT) before dispersion of photoinitiators due to their high viscosity.

### 2.3 3D Printing

A commercial 3D DLP printer (Asiga Max X UV385) with a light source centered at 385 nm was employed. For the printing procedure, the printer temperature was set at 40 °C for reducing viscosity and for operating in controlled conditions. TEGORAD and EB4740 were employed to build complex 3D structure for further investigations due to their better performances; thus, printing parameters are reported here. The detailed printing parameters found experimentally for all the photoreactive inks are listed in Table S2 in the Supporting Information. For all of the materials, the slice thickness was set at 50 μm.

With regards to TEGORAD, three burn-in layers were irradiated with a light intensity of 29 mW/cm^2^ for 2 s. For the following layers, light intensity was reduced to 26 mW/cm^2^ maintaining an exposure time of 2 s. Relatively slow approach velocity (1.2 mm/s) and separation velocity (0.5 mm/s) were chosen to avoid suction effect under the building platform due to ink viscosity.

EB4740 structures were printed setting a light intensity of 24 mW/cm^2^ and an exposure time of 1.75 s for two burn-in layer. In the subsequent layers, light intensity and exposure time were reduced to 17 mW/cm^2^ and 1.25 s, respectively. A separation velocity of 0.5 mm/s and an approach velocity of 1.2 mm/s were maintained for the whole printings. The same printing parameters were chosen for the other urethane-based resins (EB8232, EB8254, EB8411), excluding EB8413.

For multimaterial components, different ranges were set in the printing software with printing parameters optimized for each formulation envisaged. The printing procedure was then stopped at the designed layer, allowing the substitution of the printable ink, and then restarted. This allowed to have copolymerization between the different materials, avoiding the use of glues or sealing agents.

After the printing process, samples were removed from the build tray and immersed into ethanol under mild sonication (Sonorex Digiplus, Bandelin) for 3 min to remove unreacted resin residuals. Then, postcuring was performed under UV exposing the samples for 2 additional minutes (Robofactory UV).

### 2.4 Physicochemical Characterization

The viscosity of the different resins was measured at RT and 40 °C using an Anton Paar Physica MCR 302 rheometer in 25 mm diameter parallel plate mode. A gap of 1 mm between plates was set, and rotational shear ramp tests between 1 and 100 s^–1^ were performed.

The reactivity of the photocurable formulations was evaluated by real-time photorheology analysis using a 25 mm diameter parallel plate system equipped with a quartz lower plate. The tests were performed in the linear viscoelastic region according to previous tests at a constant shear frequency of 1 Hz and a constant strain amplitude of 1%. The light source used was a Hamamatsu LC8 lamp, equipped with a light guide (UV light, intensity 25 mW/cm^2^). The light was turned on after 60 s to allow for stabilization of the system. All of the rheological tests were performed at 25 °C with a gap of 0.2 mm. The variation of the polymer viscoelastic properties (*G*′*, G*′′) were followed upon light irradiation, mimicking the photopolymerization process that occurs in the VAT.

The insoluble fraction of the printed samples was determined following the standard test method ASTM D276584. The samples were weighed and held in a metal net that was immersed in chloroform for 24 h to perform the extraction of the unreacted monomers at room temperature. At last, the samples were dried overnight at 80 °C, and the insoluble fraction percentage was determined as the weight difference before and after solvent extraction. In the case of sample EB 8413 in which 50 wt % of PC was added, the amount of solvent used to decrease the viscosity was subtracted from the total initial weight.

Static contact angles of the samples were determined using an OCAH 200 contact angle system (Dataphysics Instruments, Germany), equipped with a video camera and an image analyzer (Leica DM2500). The tests were performed at room temperature using the sessile drop technique. A 3 μL droplet of deionized water (σ_d_ = 21.8 mN m^–1^, σ = 72.8 mN m^–1^) or diiodomethane (σ_d_ = 50.8 mN m^–1^, σ = 50.8 mN m^–1^, 99% purity, SIgma Aldrich) was placed onto the sample surface and the static angle was measured. Three measurements were collected for each tested sample, determining the angle by an ellipse approximation of the drop profile. More details can be seen in the Supporting Information.

Differential scanning calorimetry (DSC) measurements were performed with a DSC1 STARe system apparatus from TA Instruments equipped with a low temperature probe. The experiments were carried out between −90 and 60 °C with a heating rate of 10 °C/min.

Tensile tests were carried out using an Instron 3366 dynamometer equipped with a load cell of 500 N. Printed flat specimens (8 × 60 × 0.6 mm) were prepared, and at least three specimens for each material were tested.

### 2.5 Electromechanical Characterizations

Triboelectric testing setup was composed of a mechanical shaker (TV51110, Tira System) driven in a closed loop by a controller unit (VR 9500, Vibration Research) and a power amplifier (BAA 120, Tira System). The feedback signal was provided by an accelerometer (352C33, PCB Piezotronics) screwed onto the vibrating plate of the shaker. One layer of the triboelectric device was mounted on the shaker plate, while the other one was placed on the horizontal beam of a fixed rigid frame in order to guarantee the contact and separation steps during shaker motion. The electrical output analysis of the voltage and current was performed with a high impedance electrometer (Keithley 6517b, Tektronix).

## 3 Results and Discussion

### 3.1 Selection of Photocurable Resins

DLP printing is an intriguing production process for TENGs; nevertheless, little is known about the triboelectrical properties of light-printable materials. Thus, several photocurable acrylate monomers and oligomers were chosen and used to fabricate 3D printed specimens for defining a triboelectric series. Some of the most used monomers in DLP printable formulations were thus selected, according to the literature,^35^ namely, PEGDA,^36,37^ HDDA,^38^ and BEDA.^39,40^ Moreover, a DLP printable PDMS-like silicone acrylate (i.e TEGORAD)^41^ was selected since polydimethylsiloxane (PDMS) is known for its elevate tribolectronegativity.^42,43^ At last, commercial urethane acrylates, presenting different structures, were also evaluated (EBECRYL series), since polyurethanes have recently been proposed for the production of TENGs^44,45^and polyurethanes materials were exploited for DLP printing.^46−48^

**Figure 1:**
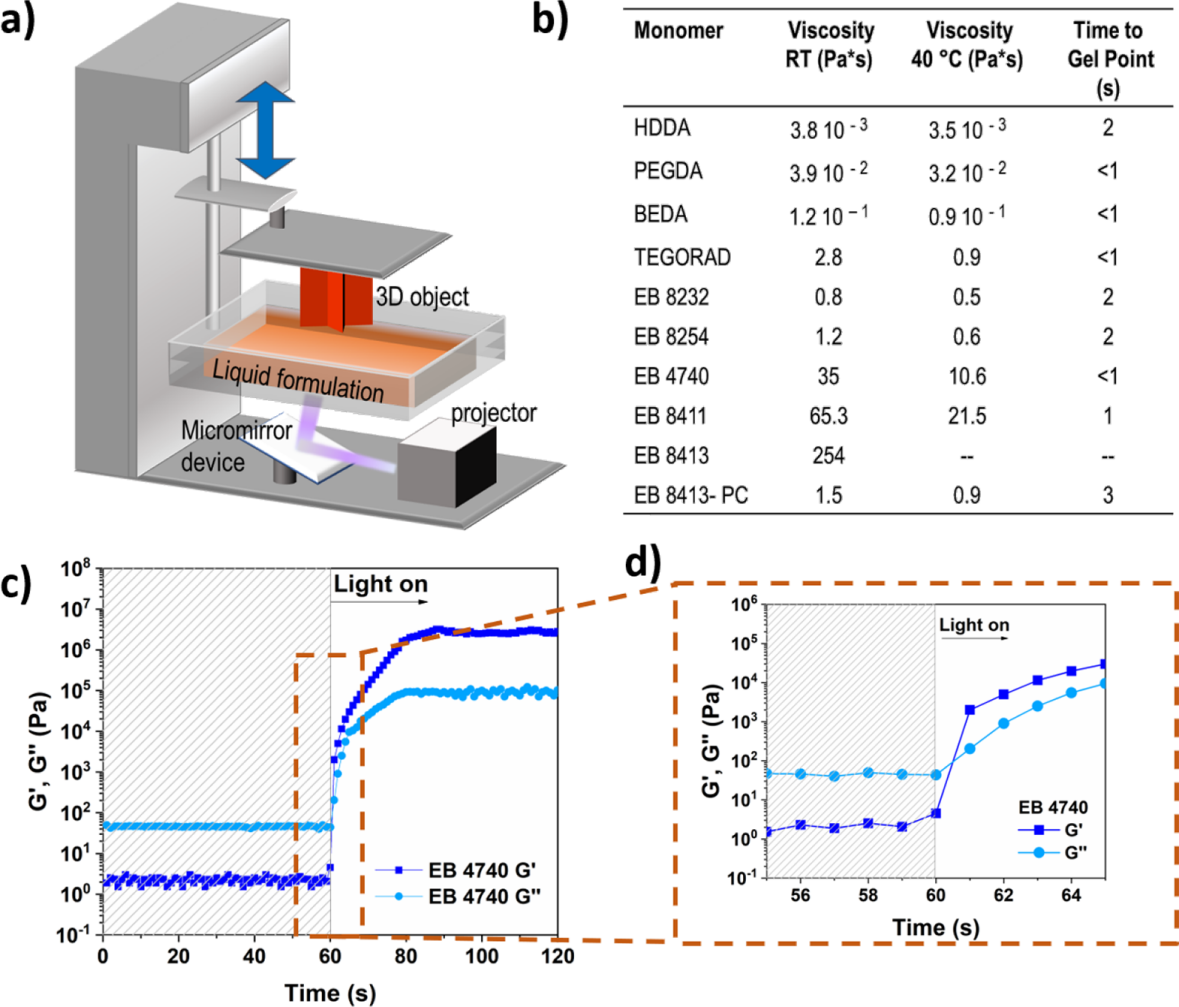
Printing properties of the photocurable resins. (a) Sketched depiction of a DLP printer. (b) Table summarizing the viscosity measured for the tested materials at room temperature and at 40 °C and the time needed to reach the gel point obtained from the photorheology measurements. (c) Photorheology plot for sample EB 4740 describing the variation of *G*′ and *G*′′ upon light irradiation; the light was turned on after 60s (UV light, 25 mW/cm^2^). (d) Magnification of the photorheology plot indicating the gel point (*G*′ = *G*′′).

In a DLP system, the light source illuminates the liquid resin vat from below through a transparent window, while a building platform is dipped into the formulation from above (bottom-up configuration, Figure 1a). To obtain the fast production of precise structures, the viscosity and reactivity of the liquid formulations and the final mechanical properties of the resins play a key role. These properties were evaluated for the selected monomers through rheological measurements. First, the viscosity of the different monomers/oligomers formulations was tested (Figure 1b and Figure S1). As expected, the monomers commonly used in DLP (HDDA, PEGDA, and BEDA) present low viscosity values at room temperature and orders of magnitude below 10 Pa·s, a value that is requested for the printing procedures.^49,50^ Differently, some of the tested urethane acrylates presented high viscosity (>10 Pa·s) and consequently, printing was performed at 40 °C, inducing a desired decrease of the viscosity. However, in the case of EB 8413, the viscosity values were still too high for printing process, and thus a solvent with high-temperature boiling point (propylene carbonate, PC) was added to achieve printability.

Despite the high viscosity of some of the selected materials, photorheology tests revealed a high reactivity for all of the compounds. To show the approach used for checking the reactivity of the resins, Figure 1c reports as an example the plots obtained for sample EB 4740 (the curves for the other formulations are reported in the Supporting Information Figure S2). The variation of the storage (*G*′) and Loss (*G*′′) moduli upon light irradiation is followed, giving important information about the reactivity of the materials. In detail, the gel point (*G*′*=G*′′) indicates the starting of the formation of the thermoset network (Figure 1d), while the high slope of the plot implies the fast reaching of the mechanical properties needed for the 3D printing process. All the tested materials showed short gelation times (Figure 1b), high slope of the curves, and final *G*′ modulus of ≫10^4^ Pa, which is usually sufficient for obtaining self-supporting structures and also for very soft materials.^50,51^ Considering the collected information on viscosity and reactivity, the printing parameters were then adjusted to obtain printed structures (see SI Table S1). The complete polymerization of the printed parts, necessary to obtain stable materials, was confirmed evaluating the amount of insoluble fraction after extraction insolvent, which was always higher than 90% (see SI, Table S2), demonstrating the formation of the cross-linked network.^31^

### 3.2 Triboelectric Series of DLP Resins

**Figure 2:**
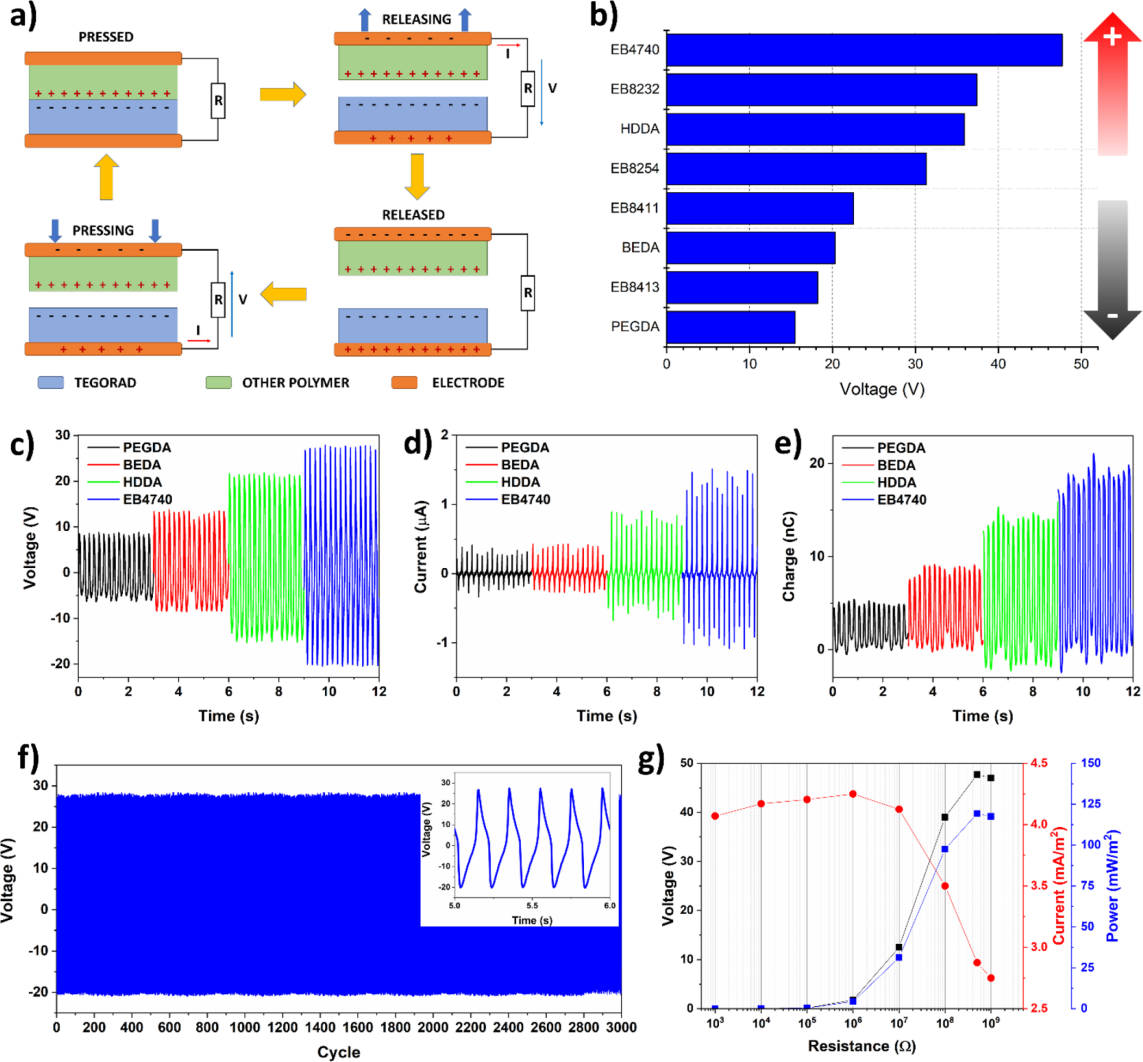
Triboelectric characterization of photocurable resins. (a) Scheme of the working principle of the contact-separation printed TENG. (b) Triboelectric series of photocurable inks for the DLP printing technique. (c) Electrical voltage, (d) current, and (e) charge output of the TENGs composed of TEGORAD as tribonegative layer and different resins as tribopositive layer. (f) Long-term performance under mechanical stimulus of the TEGORAD-EB4740 TENG. A zoom over some mechanical cycles is reported in the inset. (g) Output voltage, current, and electrical power of TEGORAD-EB4740 TENG for different load resistances ranging from 1 kΩ to 1 GΩ.

The different printed materials were then tested to compose triboelectric nanogenerators (TENGs) in contact separation mode with the aim of creating a triboelectric series of resins used. For this purpose, flat square specimens with a 2 × 2 cm^2^ area and 1 mm of thickness were printed and then coupled together on a mechanical shaker to form a triboelectric nanogenerator device. Each material generation was tested using the TEGORAD layer as negative triboelectric reference because silicone polymers are among the materials with the highest capacity of acquiring negative charges during the contact electrification process, as widely demonstrated in the literature.^42,52^ The working mechanism of the exploited TENGs, based on contact charging and electrostatic induction, is shown in Figure 2a. When an external mechanical force is applied to the TENG, the two printed layers are put in contact. At that point, charges transfer between the two materials. When the mechanical stimulus is released, an electrical potential difference is established because of the layers’ separation. This generated voltage induced charges to flow on the external load from one side to the other side to compensate for the internal TENG potential difference. This current will flow until the layers are completely released and the electrical potential is fully balanced by the charges accumulated on the electrodes. Then, when the force is applied again, the electrical potential inside the device is reduced because of the reduction of the layers’ separation. A current in the opposite direction is established to reduce the charges on the electrodes up to disappearing when the two layers return in contact and the cyclic conversion from mechanical energy to electrical energy returns to the starting point. The output signal of a TENG under cyclic mechanical loading will thus result in an alternate electrical potential and current. Putting in order the peak-to-peak voltages generated by the TENG composed of TEGORAD and the others DLP printable materials, from higher to lower, a triboelectric series of the tested DLP resins was composed (Figure 2b). The lower electrical output (15.5 V) was obtained by the TEGORAD-PEGDA couple, as also visible by voltage, current, and charge signal in Figure 2c–e. Going further in the scale from the negative triboelectric reference, the output voltage and current increase up to reach the maximum with the acrylate polyurethane EB4740 with a peak-to-peak open circuit voltage of 47.7 V, short-circuit current of 2.5 μA and output charge of 20 nC. As countercheck of the correctness of the triboelectric series built up, TENGs made of materials from intermediated position in the series were composed and measured. An example is reported in Figure S3 with the output voltage signal of a PEGDA-HDDA TENG which generation is around 10.6 V, much lower than the 35.9 V of the couple composed by HDDA and the most negative triboelectric resin TEGORAD (Figure 2b).

To evidence a possible correlation between surface properties and the triboelectric series, the surface energy was calculated following the Fowkes model,^53^according to the data obtained from the contact angle measurement (results are reported in Table S3 in the SI). Although a certain relationship seems to emerge, since the material with the highest surface (EB 4740) is also the best nanogenerator, while materials with low surface energy tend to produce lower voltages, it is not possible to define a clear trend. The relationship between physicochemical properties of the materials and tribolectric effect is still under debate,^54,55^ and in this sense, additional experiments are planned, but those are out of the scope of this study.

Coming back to the tested tribolectric series, TENG composed by TEGORAD and EB4740 represents the most performing material combination, and therefore, it was further characterized and used to print devices with a more complex shape, exploiting DLP 3D printing technology. The stability of the TEGORAD-EB4740 TENG was characterized by long-term contact-separation motion cycles as shown in Figure 2f. The device exhibits no mechanical fatigue or degradation in electrical output after 3000 cycles, evidencing excellent performance reliability for practical applications. The real maximum electrical power generation of the TENG cannot simply be evaluated by multiplying the open circuit voltage and short circuit current, but it needs to be tested measuring the induced voltage and current on an external load. The device was tested, measuring generated voltage and current on resistors ranging from 1 kΩ to 1 GΩ. As shown in Figure 2g, current output decreases with higher load, while the voltage output has an opposite behavior. The maximum output power density reached a peak value of 120 mW/m^2^ with a load resistance of 500 MΩ, with the value in line with the electrical power density generated by others 3D printed TENGs, as reported in Table S4 in the SI.^22,26,56,57^

### 3.3 3D-Printed TENG Structures

**Figure 3:**
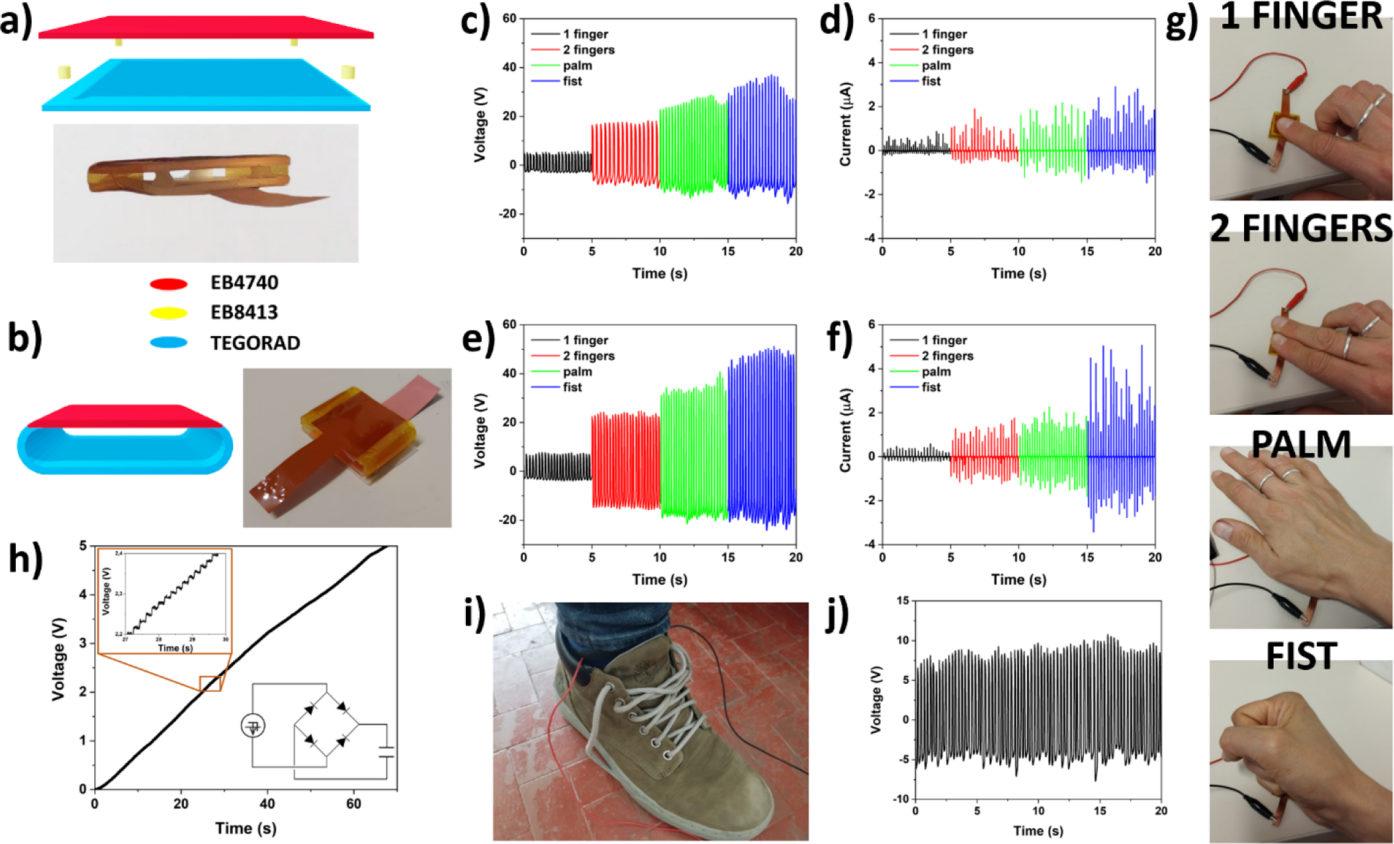
Performances of printed flat TENG. Schemes and images of the 3D printed (a) “spacers” and (b) “spring” TENG. Color legend of the different materials involved in the printing is reported. (c) Electrical voltage and (d) current output of the 3D printed “spacing” TENG upon different human mechanical movements performed with the hand. (e) Electrical voltage and (f) current output of the 3D printed “spring” TENG. (g) Images of the four different mechanical compressive stimuli applied with a human hand on the 3D printed TENGs. (h) Charging of 1 μF supercapacitor for 70 s during pressing and release with consecutive first the “spring” TENG. The inset on the top-left shows the zoom of the curve underlining the charging steps, while inset on the bottom-right shows the equivalent circuit scheme of the TENG coupled to the capacitor with a rectifier bridge. (i) Image of the “spring” TENG mounted in a shoe and (j) voltage output of the device during fast walking.

The advantages of 3D printing technology rely mostly on the capability of easily fabricating complex geometry structures. For this reason, the DLP technique was exploited to 3D print TENG devices with increasing complexity, using the results previously presented on the materials generation analysis. In particular, it is worth highlighting that DLP allows multimaterial printing in which the different layers are chemically bound, decreasing issues of adhesion and limiting delamination.^31^ The silicone acrylate TEGORAD and polyurethane acrylate EB4740 were selected as tribo-negative and tribo-positive layers, respectively, to gain the maximum electrical power output under mechanical stimuli. First, two structures operating in contact-separation mode were developed with a single step based on a consecutive multimaterial printing approach. The first TENG structure (“spacers”) was produced by printing a planar TEGORAD layer of 22 mm × 22 mm and then replacing the printable formulation with EB8413 (printed using PC solvent), to obtain four cylinders of radius and height of 1 mm in the four corners to work as separators. Finally, EB8413 was replaced with EB4740, to print the upper planar triboelectric layer of 22 mm × 22 mm. A scheme and an image of the “spacers” TENG is shown in Figure 3a. EB8413 was selected as a separator material because of its good adherence to both TEGORAD and EB 4740 and optimal elastic behavior. Indeed, the EB8413 cylinders need to guarantee a good elastic return when compressive mechanical stimulus is removed to restore the layers separtion. The second complete triboelectric nanogenerator architecture developed (“spring”) is shown in Figure 3b. In this case, first the tribonegative TEGORAD part, composed of a flat layer and lateral rounded support springs, was formed, and then printing continued by replacing the TEGORAD left in the tank with EB4740 to obtain the tribopositive flat layer. The dimensions of the flat layers are 20 × 20 mm^2^, while the lateral springs have a semicircular shape with an internal radius of 1.5 mm and an external one of 2.5 mm. The lateral springs were included in the design to guarantee the mechanical return of the layer separation under cyclic compression, exploiting the elastic properties of the silicone acrylate. Both structures can be used to harvest mechanical energy from biometric activities, which can cause cyclic compression. Examples of these applications are shown by periodically pressing TENG devices with one finger, two fingers, the hand palm, and a fist, as shown Figure 3g. Voltage and current outputs of the two printed TENG structures are reported in Figure 3c–f (charge outputs are reported in Figure S4). The lowest electrical generation was obtained cyclically pressing the TENGS with one finger since the compressed area is lower than the total device one and thus the charges generated during layer contact are less than the other conditions. Instead, the whole areas of the TENG layers come in contact while pressing the TENGs with two fingers, the palm, and the fist, generating a higher charge. When the surface of the TENG is totally compressed, the electrical output increases with the value of the compressive force applied on the device with the hand, as evidenced by force measurement shown in Figure S5. The electrical output generated by the TENG can be stored in a capacitor and later used to power portable devices. Figure 3h shows the charging behavior of a 1 μF capacitor by using the output from the “spring” TENG, after signal conditioning with a rectifier circuit. Results show that the capacitor can be charged to 5 V in around 1 min of tapping with fists, where each hit will add around 20 mV of electrical potential on the storage unit. The devices show the capability to harvest other human body mechanical movements, such as walking or running. Figure 3i–j shows the voltage generation of the “spring” TENG mounted in a shoe during fast walking.

**Figure 4:**
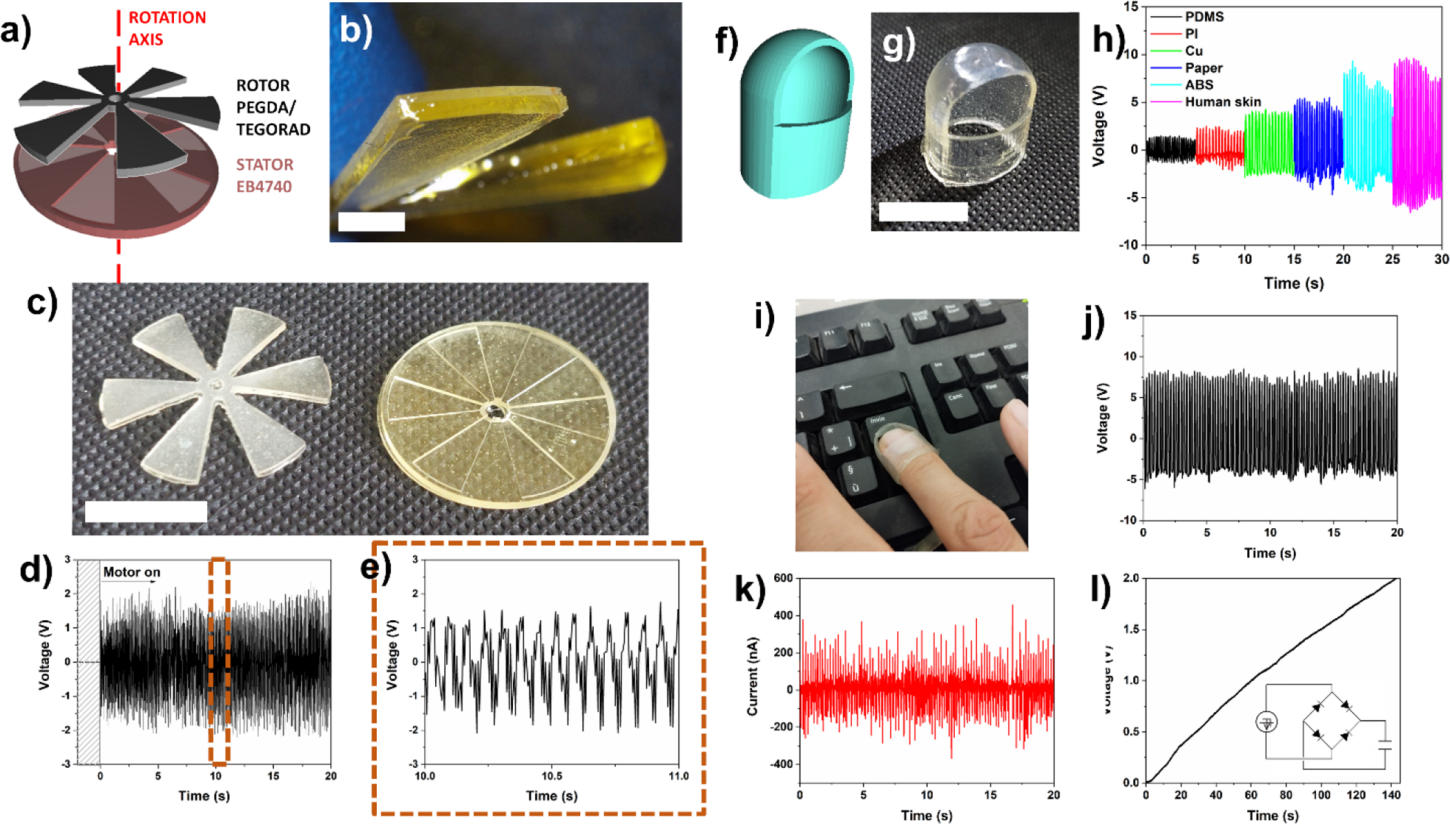
3D-printed TENG with complex geometry. (a) Scheme of the 3D-printed rotating TENG. (b) Image of the rotor blades showing the PEDGA/TEGORAD layers. Scale bar corresponds to 2 mm. (c) Image of the printed rotor and stator components of rotating TENG. Scale bar corresponds to 1 cm. (d) Electrical voltage output of the rotating TENG while switching on the DC motor providing mechanical rotation to the TENG rotor. (e) Enlarged view of the electrical voltage output showing the 14 Hz signal periodicity related to DC motor rotation. (f) CAD and (g) image of the 3D-printed thimble. (h) Electrical voltage output of the 3D-printed single electrode thimble TENG upon pressing on different materials. (i) Image of the TENG mounted on the tip of the thimble while pressing the enter key of a keyboard. (j) Electrical voltage and (k) current output of the TENG during periodic pressing of the enter key. (l) Charging of 1 μF supercapacitor for 140 s during keyboard typing. Inset shows the equivalent circuit scheme of the TENG coupled to the capacitor with a rectifier bridge.

3D printing approaches enable the fabrication of more intricate TENG structures than those achievable through traditional casting techniques, as has been demonstrated with other additive manufacturing techniques.^20,22,24,58^ Therefore, here, the DLP approach was employed to print more complex structures, showcasing its versatility and potential. For instance, a rotating TENG designed to harvest mechanical energy using the lateral sliding principle was prepared. A system composed of a fixed stator and a moving rotor was printed as shown in Figure 4a–c. The stator was printed in EB4740, while the rotor was fabricated with the multimaterial printing approach of PEGDA and TEGORAD. TEGORAD is a soft material and would not have the rigidity necessary to compose the stator; therefore, TEGORAD was printed on a more rigid PEGDA layer (as shown in Figure 4b), which guarantees the needed mechanical properties; glass transition temperature (*T*_g_) values and elastic moduli of the selected materials are reported in Table S5 (SI). With this approach, both the rotor and stator would have a good rigidity to sustain high speed rotation, while the sliding and charging generation process was guaranteed through the contact between TEGORAD and EB4740. The rotor part of the TENG was connected to a DC motor, which provides the rotation movement. The TENG was able to generate electrical voltage when DC motor was powered up (Figure 4d), because of the sliding process happening between the blades of stator and rotor. The peak-to-peak output voltage was around 4 V and follows the rotating movement of the DC motor at around 14 Hz (Figure 4e). The voltage output is lower than contact-separation TENGs showed before, because of the different physical contact between the two triboelectric layers. Nevertheless, these results demonstrate that the DLP printing technique could be used to fabricate several types of triboelectric nanogenerators and it is not limited to a single working modality.

Lastly, an example of a single electrode triboelectric nanogenerator was fabricated with DLP technology, exploiting the great flexibility of the techniques in printing complex geometry devices. A TENG with thimble geometry was printed in TEGORAD to exploit the elevate tribolectronegativity of the photocurable resin, as shown in Figure 4f,g; in this case, the good flexibility of TEGORAD also guaranteed a good conformability. A flexible copper-polyimide electrode connected to the electrical ground was placed to the internal part of the thimble to mount the TENG. Contact electrification of TEGORAD happens when the thimble is fully in contact with an external material and then, when it is released, electrons are exchanged with the ground to balance the generated potential difference between TEGORAD and ground until equilibrium is reached. Subsequently, a reversed electron flow arises when the thimble is approached back for contact. Therefore, the thimble could generate electrical voltage when pressed on a different material and then released depending on the triboelectric properties of the materials and their position in the triboelectric series. The TENG was inserted in the ring finger and tested, keeping as much as possible unaltered the pressing force, on PDMS, polyimide (PI), copper, paper, acrylonitrile butadiene styrene (ABS), and human skin. The materials were ordered going from the negative to the positive part of the triboelectric series for standard material, and, as expected, the electrical peak-to-peak output voltage generated by the thimble TENG increased from 2.5 to 15 V (Figure 4h). The TENG was then used to prove its implementation in an everyday activity like typing on a keyboard, made of ABS polymer (Figure 4i). The thimble TENG generated electrical voltage and current during periodic pressing of the enter key (Figure 4j,k). While pressing the key, the TENG was also able to charge a 1 μF capacitor up to 2 V in 140 s, upon connection to a rectifier circuit (Figure 4l). TEGORAD material is highly flexible and elastic; thus, no mechanical damage to the thimble is observed during the capacitor charging procedure. These results demonstrate the capability of the thimble-printed TENG to harvest human mechanical movements and envision the possibility of using the device, after a proper calibration, as a sensor to evaluate the touched material.

## 4 Conclusions

In conclusion, some photocurable resins for DLP printing were tested as triboelectric layers in a contact-separation triboelectric nanogenerator configuration. Depending on their tendency to accumulate electrical charges on the surface upon friction, the materials were organized in a triboelectric series with TEGORAD, a silicone acrylate resin, being the most tribonegative material, and EB4740, a polyurethane acrylate, the most tribopositive one. The best performing couple of materials were used to fabricate 3D-printed TENGs devices with increasing geometrical complexity using a multimaterial printing procedure. Contact-separation flat TENGs, spinning rotor TENG, and a single electrode thimble TENG were produced, and their capability of harvesting mechanical energy from human movements converting it into electrical energy was demonstrated. The results presented in this work illustrate how the DLP approach can be used to fabricate complex triboelectric nanogenerator able to recover energy from different kinds of mechanical movements and, most of all, provides a triboelectric series of the most common photocurable resins, which will serve as a solid foundation for developing further investigations of triboelectric nanogenerator devices fabricated with this highly flexible printing method.

## Supporting Information Available

List of the chemicals used and relevant information; printing parameters for the different formulations; viscosity and photorheology measurements; voltage output of the TENG used as control for establishing the triboelectric series; contact angle measurements and calculation of surface energies of the polymers used; comparison between this work and the output power density performances of some of 3D printed TENG devices presented in the literature; charge output of 3D-printed specimens subjected to different movements; loads measured for different human movements; and glass transition temperature and Young’s modulus of the materials used in 3D-printed devices (PDF)
