# Supplementary material for: Three-Dimensional Printing of Triboelectric Nanogenerators by Digital Light Processing Technique for Mechanical Energy Harvesting
Source: ACS Appl Mater Interfaces. 2023 Nov 9;15(46):53974–83. doi: 10.1021/acsami.3c13323 (PMC10685350; doi:10.1021/acsami.3c13323)
Supplement: Supplementary file 1 — am3c13323_si_001.pdf [file am3c13323_si_001.pdf]

SUPPORTING INFORMATION

3D printing of triboelectric nanogenerators by Digital Light Processing technique for mechanical energy harvesting

Annalisa Chiappone[a], Ignazio Roppolo[b,c], Edoardo Scavino[b], Giorgio Mogli[b], Candido Fabrizio Pirri[b,c], Stefano Stassi[b]*

a) Department of Chemical and Geological Sciences, Università degli studi di Cagliari, Cittadella Universitaria Blocco D, S.S. 554 bivio per Sestu 09042 Monserrato, CA, Italy

b) Department of Applied Science and Technology, Politecnico di Torino, C.so Duca degli Abruzzi 24, 10129 Turin, Italy

c) Center for Sustainable Future Technologies @Polito, Istituto Italiano di Tecnologia, Via Livorno, 60, 10144 Turin, Italy

** Corresponding author: ignazio.roppolo@polito.it*

.

Table S1 Chemical composition of the monomers used in this work.

	Chemical structure	Characteristics
**TEGORad 2800**	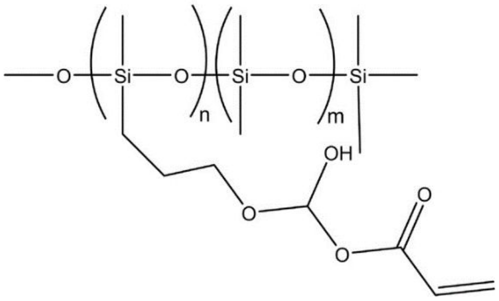	Silicone acrylate, molecular weight undisclosed
**EB4740**	n.a.	Trifunctional aliphatic allophanate urethane acrylate, chemical formula undisclosed
**EB8254**	n.a.	Hexafunctional aliphatic urethane acrylate, chemical formula undisclosed
**BEDA**	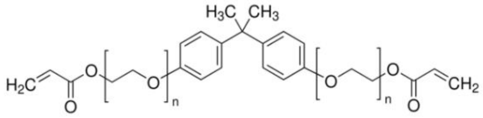	Molecular weight 512 Da, ratio EO/Phenol 2:1
**HDDA**	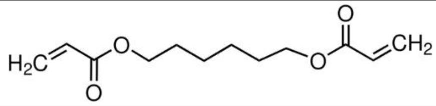	Molecular weight 512 Da
**EB8232**	n.a.	Difunctional aromatic urethane acrylate, chemical formula undisclosed
**EB8413**	n.a.	Urethane Acrylates, chemical formula undisclosed
**PEGDA**	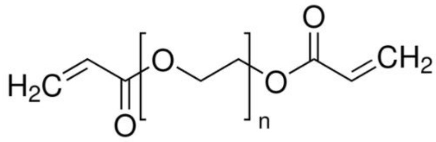	Mn 700 Da
**EB8411**	Urethane component<br>N.a.<br>IBOA<br>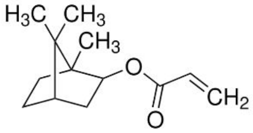	Aliphatic urethane diacrylate diluted 20% by weight with the reactive diluent isobornyl acrylate (IBOA), chemical formula of the urethane component undisclosed

Table S2 Guidelines of printing parameters for the analysed inks and insoluble fraction measured on the printed samples. All the printings were performed at 40°C with a slice thickness of 50 µm. Burn-in layers were set to facilitate adhesion of the 3D structure to the build tray. In the last column, the insoluble fraction percentage for the analysed materials is reported.

Formulation	N° burn-in layers	Light intensity (mW/cm^2)		Exposure time (s)		Separation velocity (mm/min)		Approach velocity (mm/min)		Wait time after each step (s)		Insoluble fraction (%)
		Burn-in	Others	Burn-in	Others	Burn-in	Others	Burn-in	Others	Burn-in	Others	
**TEGORAD**	3	29	26	2	2	0.5	0.5	1.2	1.2	1.3	1.3	94
**PEGDA**	2	33	28	1.25	1	3	3	3	3	0.75	0.75	94
**BEDA**	1	28	26	6	4	0.6	0.6	1.1	1.1	0.5	0.5	92
**HDDA**	3	30	25	2	1.75	3	3	3	3	0.75	0.75	99
**EB4740**	2	24	17	1.75	1.25	0.5	0.5	1.2	1.2	3	3	97.5
**EB8232**	2	24	17	1.75	1.25	0.5	0.5	1.2	1.2	3	3	99
**EB8254**	2	24	17	1.75	1.25	0.5	0.5	1.2	1.2	3	3	98
**EB8411**	2	24	17	4	2	0.5	0.5	1.2	1.2	3	3	92
**EB8413**	2	49	49	15	15	0.5	0.5	3	3	1	1	90

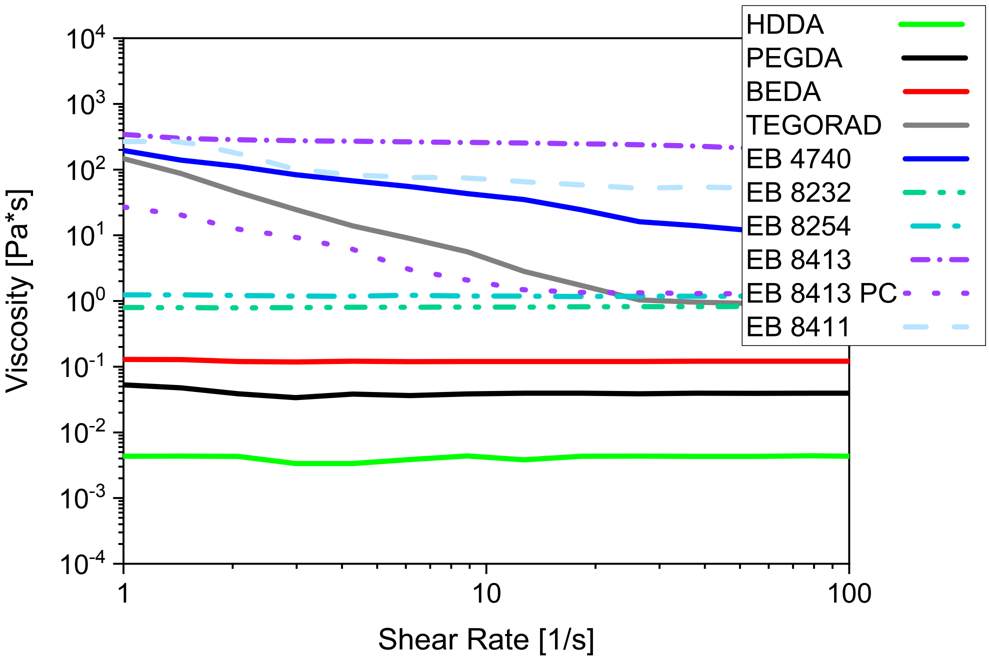

Figure S1. Viscosity vs shear rate measured at room temperature.

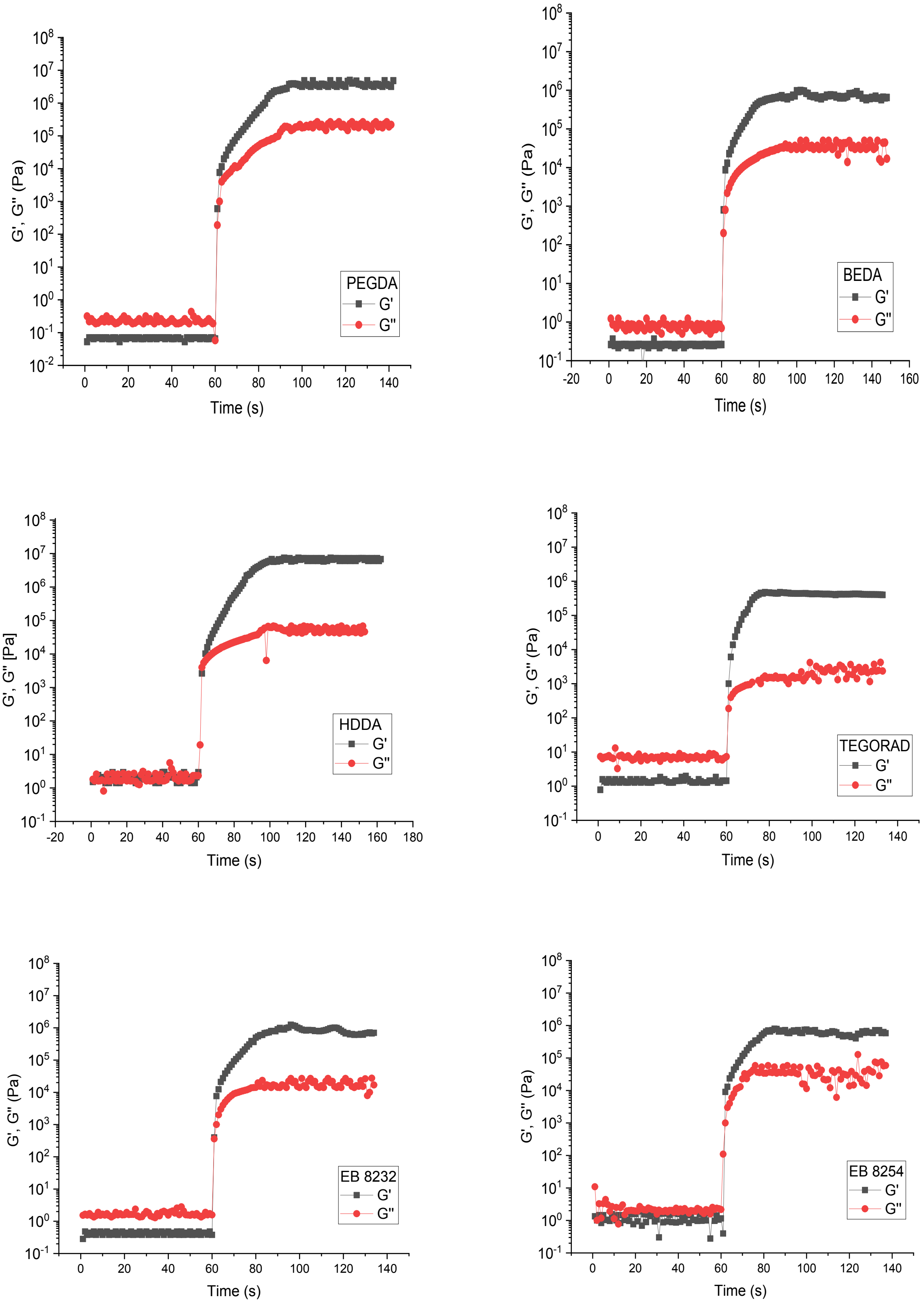
PEGDA
G'
G"
G', G" (Pa)
Time (s)
BEDA
G'
G"
G', G" (Pa)
Time (s)
HDDA
G'
G"
G', G" [Pa]
Time (s)
TEGORAD
G'
G"
G', G" (Pa)
Time (s)
EB 8232
G'
G"
G', G" (Pa)
Time (s)
EB 8254
G'
G"
G', G" (Pa)
Time (s)

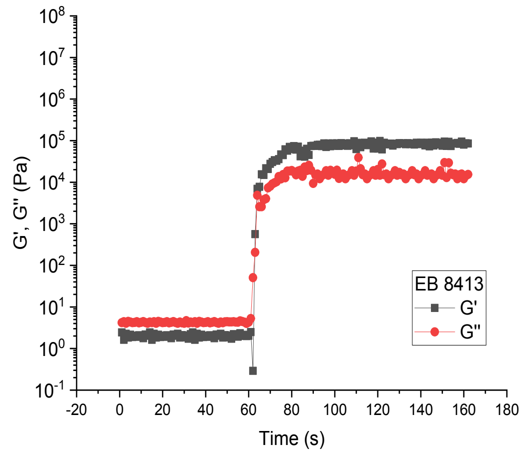

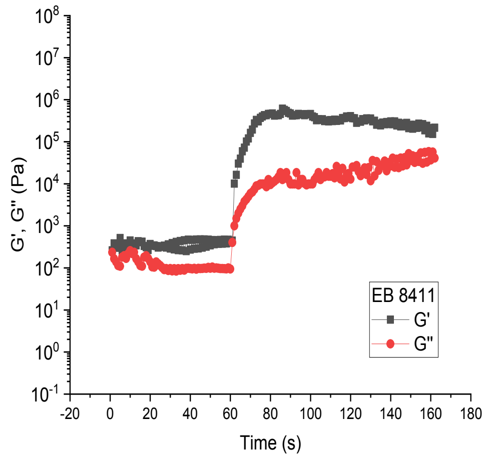

Figure S2. Photohreology plots for the different formulations

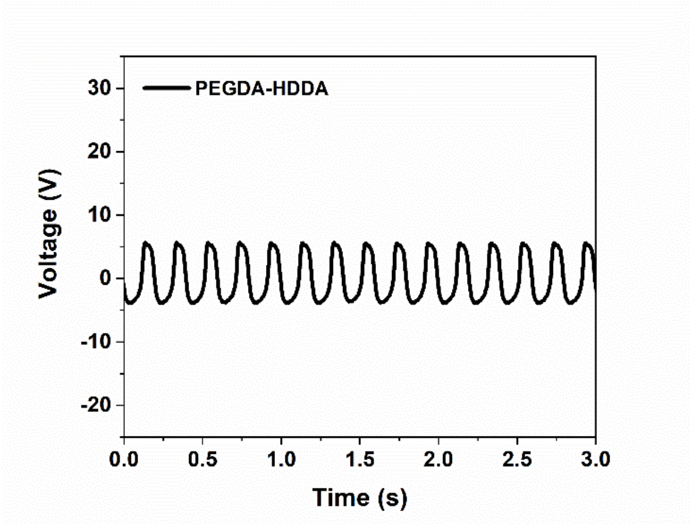

Figure S3. Voltage output of the TENG composed of PEGDA and HDDA used as control for the triboelectric series.

Table S3 Contact angles measured with water and Di-iodomethane, calculation of the apolar and polar components and surface tension.

	**$\theta(H_2O)$**	**$\theta(CH_2I_2)$**	**Dispersive Component** (mN/m)	**Polar Component** (mN/m)	**Surface energy** (mN/m)	**Position in the triboelectric serie**
**TEGORad**	106.7	64.3	22.12	49.89	72.02	X
**EB4740**	69.6	28.7	0.10	86.73	86.83	1
**EB8254**	100.8	60.6	3.10	78.49	81.59	4
**BEDA**	71.3	31.9	65.71	10.74	76.45	6
**HDDA**	92.5	50.6	68.82	1.50	70.32	3
**EB8232**	51.4	57.0	64.66	3.97	68.63	2
**EB8413**	104.7	74.1	29.32	0.87	30.19	7
**PEGDA**	64.9	52.1	9.95	0.57	10.52	8
**EB8411**	104.3	77.3	8.29	1.02	9.31	5

For the calculation of the surface tension Fowkes model was used, according to the following equation:

$$\sqrt{\sigma_l^D \cdot \sigma_s^D} + \sqrt{\sigma_l^P \cdot \sigma_s^P} = \frac{\sigma_l\,(1+\cos\theta)}{2} \qquad (1)$$

Where σ_s^D and σ_s^P are the dispersive and polar components of the surface energy of the solid respectively and σ_l^D and σ_l^P are the dispersive and polar components of the surface tension of the liquid respectively.

Diiodomethane was used since it has no polar component σl^P to its surface tension (due to molecular symmetry), meaning $\sigma_l = \sigma_l^D$ = 50.8 mN/m. Allowing the calculation of σ_s^D:

$$\sigma_s^D = \frac{\sigma_l\,(1+\cos\theta)^2}{4} \qquad (2)$$

The second measurement was performed with water, where σ_l^P = 51.0 mN/m and σ_l^D = 21.80 mN/m. By inserting these values into Eq.1, along with surface tension and previously calculated σ_s^D, the value of σ_s^P can be calculated. The final surface energy value is then a sum of the surface energy components.

Table S4. Comparison of the output power density performances of some of 3D printed TENG devices presented in the literature.

REFERENCE	MATERIALS	3D PRINTING TECHNIQUE	OUTPUT POWER DENSITY
THIS WORK	TEGORad, EBECRYL 4740	DLP	120 mW/m^2
[1]	Nylon, Aluminium	FFF	660mW/m^2
[2]	Poly(glycerol sebacate) (PGS) and carbon nanotubes (CNTs)	DIW	5.55 mW/m^2
[3]	Nickel and PTFE particles in Dragon Slow Skin 10 (DSS10)	DIW	36 mW/m^2
[4]	Cellulose, PDMS	DIW	29 mW/m^2
[5]	Silicone, Aluminium	DIW	608 mW/m^2
[6]	ABS, PTFE	DLP	1.4 mW/m^2
[7]	Polyurethane acrylate, Latex	FFF	400 mW/m^2

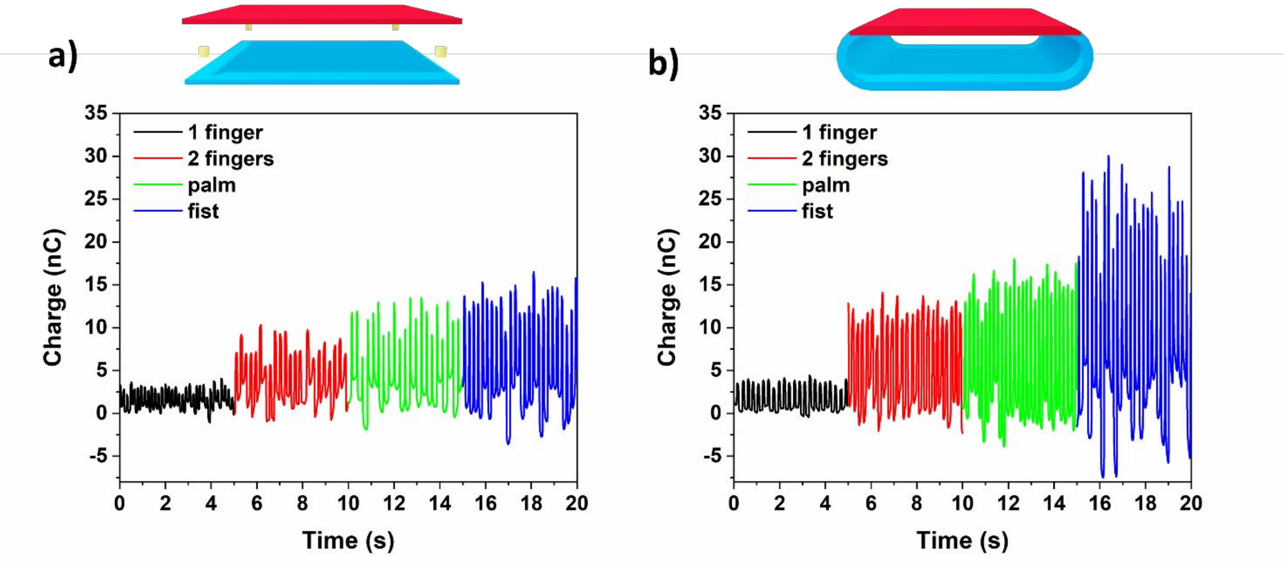

Figure S4. Charge output of the 3D printed a) “spacers” and b) “spring” TENG upon different human mechanical movements performed with the hand.

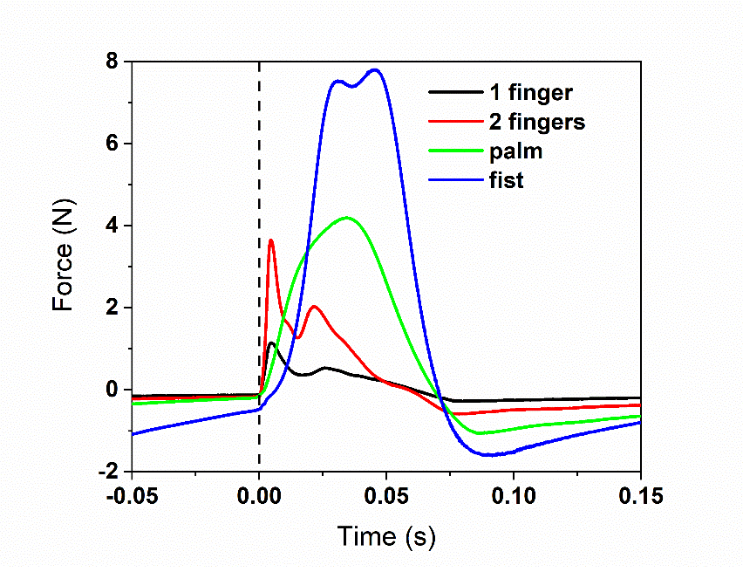

Figure S5. Force measured with a load cell applied below “spring” TENG upon different human mechanical movements performed with the hand.

Table S5. Glass Transition temperature and Young’s modulus of the materials used for the printing of the 3D TENGs

	Tg (°C)	E (MPa)
**TEGORAD**	-45	1
**PEGDA**	-40	19
**EB 4740**	50	292

REFERENCES

[1] M.-L. Seol, R. Ivaškevičiūtė, M.A. Ciappesoni, F.V. Thompson, D.-I. Moon, S.J. Kim, et al., All 3D printed energy harvester for autonomous and sustainable resource utilization, Nano Energy, 52(2018) 271-8.

[2] S. Chen, T. Huang, H. Zuo, S. Qian, Y. Guo, L. Sun, et al., A Single Integrated 3D-Printing Process Customizes Elastic and Sustainable Triboelectric Nanogenerators for Wearable Electronics, Adv Funct Mater, 28(2018) 1805108.

[3] G. Liu, Y. Gao, S. Xu, T. Bu, Y. Xie, C. Xu, et al., One-stop fabrication of triboelectric nanogenerator based on 3D printing, EcoMat, 3(2021) e12130.

[4] C. Qian, L. Li, M. Gao, H. Yang, Z. Cai, B. Chen, et al., All-printed 3D hierarchically structured cellulose aerogel based triboelectric nanogenerator for multi-functional sensors, Nano Energy, 63(2019) 103885.

[5] H. Li, R. Li, X. Fang, H. Jiang, X. Ding, B. Tang, et al., 3D printed flexible triboelectric nanogenerator with viscoelastic inks for mechanical energy harvesting, Nano Energy, 58(2019) 447-54.

[6] H.-J. Yoon, D.-H. Kim, W. Seung, U. Khan, T.Y. Kim, T. Kim, et al., 3D-printed biomimetic-villus structure with maximized surface area for triboelectric nanogenerator and dust filter, Nano Energy, 63(2019) 103857.

[7] K. Parida, G. Thangavel, G. Cai, et al., Extremely stretchable and self-healing conductor based on thermoplastic elastomer for all-three-dimensional printed triboelectric nanogenerator. Nat Commun, 10(2019) 2158.
